# The Role of Chromatin in Adenoviral Vector Function

**DOI:** 10.3390/v5061500

**Published:** 2013-06-14

**Authors:** Carmen M. Wong, Emily R. McFall, Joseph K. Burns, Robin J. Parks

**Affiliations:** 1 Regenerative Medicine Program, Ottawa Hospital Research Institute, Ottawa, Ontario, K1H 8L6, Canada; E-Mails: cawong@ohri.ca (C.M.W.); emcfall@ohri.ca (E.R.M.); josburns@ohri.ca (J.K.B.); 2 Department of Biochemistry, Microbiology and Immunology, University of Ottawa, Ottawa, Ontario K1H 8M5, Canada; 3 Department of Medicine, University of Ottawa, Ottawa, Ontario K1H 8M5, Canada

**Keywords:** adenovirus, chromatin, histones, DNA replication, transcription

## Abstract

Vectors based on adenovirus (Ad) are one of the most commonly utilized platforms for gene delivery to cells in molecular biology studies and in gene therapy applications. Ad is also the most popular vector system in human clinical gene therapy trials, largely due to its advantageous characteristics such as high cloning capacity (up to 36 kb), ability to infect a wide variety of cell types and tissues, and relative safety due to it remaining episomal in transduced cells. The latest generation of Ad vectors, helper‑dependent Ad (hdAd), which are devoid of all viral protein coding sequences, can mediate high-level expression of a transgene for years in a variety of species ranging from rodents to non-human primates. Given the importance of histones and chromatin in modulating gene expression within the host cell, it is not surprising that Ad, a nuclear virus, also utilizes these proteins to protect the genome and modulate virus- or vector‑encoded genes. In this review, we will discuss our current understanding of the contribution of chromatin to Ad vector function.

## 1. Introduction

The biology of human adenovirus (Ad) has been studied in great detail for over 60 years, making Ad one of the best characterized human DNA viruses. These studies, while giving us a great deal of information about DNA replication, control of gene expression, and tumorigenesis, also laid the foundations for the later development of Ads as gene transfer vectors. The first suggestion that Ad could be used as a vector system for expression of foreign genes came in the late 1970s with the identification of spontaneous recombinants between Ad and SV40 which expressed T antigen fused to an Ad structural protein [1,2]. Use of this vector allowed for the purification of large quantities of a T antigen-related protein that retained biological activity and set the stage for further development of Ad as a gene delivery vehicle. A crucial development occurred when cell lines that could complement viruses deleted of the essential early region 1 (E1) were created [3]. This cell line allowed for the development of Ad-based vectors that could not replicate in most cell lines or tissues, but could provide high level expression of an exogenous transgene. Ad vectors now come in a variety of “generations” that differ in their degree of attenuation, from loss of one gene to deletion of all viral protein coding sequences. These vectors can serve a variety of functions ranging from short-term, high-level expression of a transgene in tissue culture to long-term therapeutic gene expression in animal models of human disease. 

One common feature of all Ad vectors is that they remain episomal in the transduced cell and, in some animal models, the vector can persist and express a transgene for several years. This finding suggests that the vector may be able to “hide” in the nucleus through adopting a structure similar to the genomic DNA. Thus, the chromatinized viral DNA is likely impacted and influenced by all of the same factors as the native cellular DNA, such as epigenetic regulation. Indeed, recent studies have shown this to be the case, as Ad-based vector DNA associates with histones and is wrapped in nucleosomes within the host cell nucleus. This chromatinized structure strongly influences the level and duration of expression from a vector-encoded transgene. This review will discuss our current understanding of Ad vector chromatinization. For a more detailed review of the dynamic changes in nucleoprotein structure during wildtype Ad (AdWT) infection, please see [4].

## 2. Ad Vector Design

**Figure 1:**
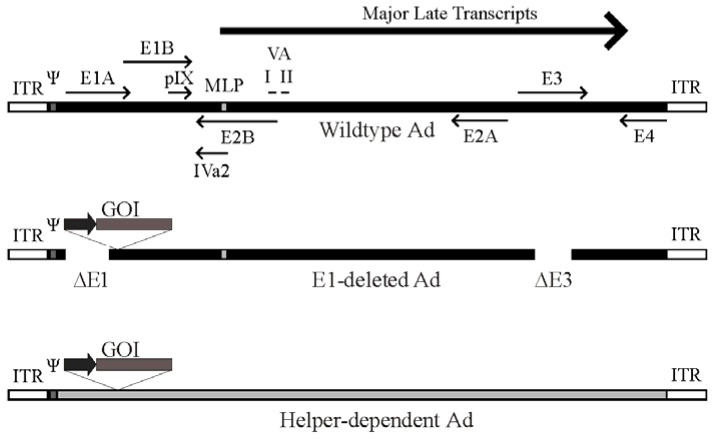
Schematic of the adenovirus genome and adenovirus-based vectors. Top panel: A simplified map of the adenovirus (Ad) serotype 5 genome showing the early genes (E1–E4) and the region from which the major late transcript is produced (the L1-L5 transcripts produced from alternative splicing of the major late transcript are not shown). The relative position of pIX, VA RNA I and II and IVa2 are indicated. Also shown are the viral inverted terminal repeats (ITR) located at each end of the genome, the viral packaging element (Ψ) located adjacent to the left ITR, and the position of the major late promoter (MLP). Please note that these features are not drawn to scale. Middle panel: General structure of an early region 1 (E1)-deleted Ad vector. Most E1-deleted vectors are also deleted of the E3 region, which is not required for replication in tissue culture and increases the cloning capacity to approximately 8 kb of foreign DNA. The gene of interest (GOI) is usually introduced to replace the E1 region and is placed under control by a heterologous promoter (dark arrow). Bottom panel: General structure of a helper-dependent Ad vector. hdAd are devoid of all protein coding sequences, and need contain only the viral inverted terminal repeats and packaging element. hdAd also frequently contain non-coding stuffer DNA (shown in gray) to ensure optimal genome size.

Although there are many different generations of Ad vectors which differ in the extent to which the genome is attenuated (reviewed by [5]), the vast majority of studies involving Ad vectors use the simple E1-deleted, first-generation Ad vector (Figure 1). Most first-generation vectors are also deleted of the E3 region, which is not required for virus replication in culture, and its removal increases the cloning capacity for foreign DNA to ~8 kb [6]. Since E1 is absolutely required for virus replication, these vectors do not replicate to any appreciable degree in most cell lines, but can be easily propagated in E1-complementing cell lines such as 293 cells [3]. However, it is important to note that the E1‑deleted Ad vectors can undergo limited replication in a number of tissue culture cell lines if they are delivered at a high multiplicity of infection (MOI) [7]. Moreover, first generation Ad delivery to animals and humans is typically accompanied by the induction of very strong innate and adaptive immune responses [8,9]. These immunogenic responses are at least in part due to leaky expression of viral genes retained in the E1-deleted vectors [10], which ultimately limits the duration of transgene expression to a few days or weeks. First generation Ad vectors are usually ideal for short-term expression in tissue culture or animals, but not ideal for applications requiring long-term (months to years) expression. Second generation Ad vectors have further deletions in the E2 or E4 coding sequences. E2 encodes proteins involved in Ad DNA replication [11], so its removal will prevent replication and reduce the expression of late genes [12]. E4 encodes several proteins involved in a variety of functions that impact on both viral and cellular gene expression and signal transduction [11]; thus, removal of some or all of E4 should significantly attenuate the vector. Deletions of E2 and E4 have been shown in some studies to have beneficial effects in reducing the expression of viral proteins and vector-directed immune responses, thereby extending transgene expression [5]. However, second generation vectors are no longer in common use due to limited evidence of enhanced efficacy over first generation vectors.

Deletion of all viral protein coding sequences results in third-generation Ad vectors, also known as fully-deleted, gutted, or helper-dependent Ad (hdAd) vectors (Figure 1). This latter term arose from the fact that these vectors must be propagated in the presence of a second virus that provides all of the replication and packaging functions *in trans* for growth of the hdAd vector [13]. The hdAd vector needs only the *cis* activating elements required for virus DNA replication (the inverted terminal repeats [ITR], ~100 bp located at both ends) and the packaging sequence (~150 bp, located immediately adjacent to the left ITR). Selection against packaging of the helper virus genome is important to ensure efficient hdAd propagation. Selective packaging can be accomplished using loxP or FRT sites flanking the helper virus DNA packaging element and 293-based cell lines expressing the Cre or FLP recombinase, respectively [13,14,15,16,17]. Such helper viruses can replicate and produce all viral proteins necessary for replication and packaging of the hdAd, but cannot package their own genomes due to loss of the packaging element in the Cre- or FLP-expressing cells. These methods result in relatively pure stocks of hdAd. In numerous studies, we and others showed that hdAd have many desirable characteristics for gene therapy applications, including long term therapeutic gene expression (>2 years) in mice [18,19,20], rats [21], dogs [22] and baboons [23], and reduced toxicity relative to traditional Ad vectors [24,25]. For a more comprehensive review of hdAd vector function, please see [5,26,27,28].

## 3. Ad Virion Structure

Although these Ad vectors lack some (or all) viral coding sequences, the overall virion structure appears identical to AdWT [29,30]. The Ad virion has an icosohedral, non-enveloped capsid structure (~70 to 100 nm in diameter) surrounding a nucleoprotein core containing a linear double-stranded genome that for wildtype Ads is ~30–40 kb. The Ad capsid is composed of 8 polypeptides, named in order of decreasing size [31,32,33,34]. Hexon (a trimer of protein II) assemble into a sheet-like structure called the “group-of-nine”, which forms the 20 facets of the icosahedon. Protein III clusters into groups of five (known as pentons) at the vertices of the icosahedron, from which extend trimers of protein IV, known as fibre. These three polypeptides are the major capsid proteins. This general structure is supported by five minor capsid proteins (IIIa, IVa2, VI, VIII, and IX). Within the viral capsid, the viral DNA associates with three basic proteins, VII, V and Mu (μ), which function to neutralize the charge on the DNA, permitting tight packing of the DNA within the virion. Protein VII is similar to cellular protamines, and is the main protein responsible for wrapping and condensing the viral DNA [35]. A shell of protein V is postulated to coat the protein VII-DNA complex [36,37]. Pre‑Mu is thought to aid in wrapping and condensing the viral DNA [38]. It is believed that cleavage of pre-Mu by the Ad-encoded protease may function to relax the viral DNA nucleoprotein structure before it enters the nucleus [39]. Since the tightly packed DNA structure is refractory to viral transcription [40,41], the nucleoprotein structure of the Ad DNA must be remodeled to achieve efficient gene expression.

Even though the viral DNA does not directly interact with the outer capsid proteins, it still contributes to the physical stability of the virion, as packaging of subgenomic sized DNA results in decreased Ad virion stability [30,42]. There seems to be a direct relationship: the smaller the genome, the less stable the virion. This observation has significant consequences for the design of Ad-based vectors. In the case of hdAd, which lacks all viral protein coding sequences, the viral genome must be replaced with alternative DNA simply to provide the necessary structural stability to support the mature capsid structure. For hdAd, reducing the genome size to less than ~27 kb causes the DNA to multimerize or rearrange to increase the genome size to between 27 and 36 kb [43]. Thus, if the transgene is large enough (e.g., a genomic loci), it may be sufficient to provide the needed support; however, a small transgene cassette may require the use of a non-coding “stuffer” element to bring the overall genome to an appropriate size. However, the nature of the stuffer DNA can significantly influence the function of the hdAd vector due to different epigenetic modifications, as discussed below [44,45].

## 4. Ad Infection of a Cell

The most commonly used Ad vectors are based on human Ad serotypes 2 or 5, and many of the details of the infection process have been worked out in great detail. Initially, the Ad fibre protein binds to the Coxsackie-Adenovirus receptor (CAR—a common receptor for Ad5 and Coxsackie B virus) [46,47]. Ad5 can also enter cells using heparin sulfate proteoglycans as an alternative receptor, either through direct binding to the Ad fibre shaft [48], or bridged through interaction of Ad with blood factors such as factor IX, factor X or complement component C4-binding protein [49,50,51]. Binding is followed by interaction between Ad penton and α_v_β_3_ or α_v_β_5_ integrins [52]. Ad is internalized by receptor-mediated endocytosis and escapes from the early endosome [53,54]. The virion is then transported through the cytoplasm to the nucleus along the microtubule network [53], and the capsid is slowly disassembled *en route* [55]. Upon reaching the nuclear pore, the Ad DNA complexed with protein VII is released into the nucleus [55,56,57], while the rest of the capsid proteins are ultimately degraded. For a more comprehensive discussion of viral entry, please see [58].

## 5. Ad Vector DNA Remains Primarily Episomal within the Infected Cell Nucleus

All vector systems have at least some ability to integrate into the genome of the host cell. Obviously, retrovirus- and lentivirus-based vectors integrate at very high frequencies in the target cell genome, making them ideal for studies involving rapidly dividing cell types (e.g., stem cells), but this does raise the risk of insertional activation or inactivation of cellular genes [59,60,61]. For Ad, the spontaneous integration rate is very low. In tissue culture studies, infection of several different cell lines with E1-deleted or hdAd vectors containing a selectable marker led to an integration frequency of 10^−4^ to 10^−5^ per cell (using an initial MOI of 10), and integration appeared to be due to non‑homologous recombination [62,63]. A similar frequency was observed for hdAd in mouse hepatocytes *in vivo* [64], suggesting the vast majority of expression from hdAd in animals is due to expression from episomal vectors. Furthermore, in infected mouse hepatocytes *in vivo*, the vectors do not replicate with the cellular DNA and remain monomeric [65]. Traditional wisdom has held that in dividing tissues, non-integrated Ad DNA would be “lost” from the nucleus of transduced cells during cell division, as the virus has no known means of segregating with the host chromosomes. However, *in vivo* studies in which hdAd-transduced mouse hepatocytes were induced to divide through partial hepatectomy showed that Ad has a surprising ability to be retained in daughter cell nuclei after division, suggesting the virus may physically associate with chromosomes in the nucleus through an uncharacterized mechanism [66,67].

## 6. Early Events within the Infected Cell Nucleus

Much of what we know about the early stages of Ad DNA remodeling comes from studies of AdWT infection, though we assume that many of these events are similar for Ad vectors. Even though many Ad capsid proteins translocate to the periphery of the nucleus, only the VII-DNA complex enters the nucleus through the nuclear pore with the help of histone H1 (H1) [56,68]. This role of H1 appears independent of any known function in eventually condensing the viral DNA in the nucleus. Studies involving UV cross-linking of radiolabeled proteins to the AdWT DNA suggest that protein VII remains stably associated with the Ad DNA throughout the early phase of infection, from at least 2 until 13 hours post infection (hpi) [69]. Several immunofluoresence-based studies using antibody directed to protein VII have shown that protein VII-containing foci can be detected in the nucleus of both AdWT- and Ad vector-infected cells, which represent protein VII-wrapped genomes [70,71,72,73]. For AdWT, these foci remain visible for at least 8–10 hpi, but disappear around 12–16 hpi [71]. Treatment of cells with alpha-amanitin, an inhibitor of transcription, prevented loss of VII foci at late times (16 hpi), suggesting that late transcription could be involved in release of VII [70,71]. However, using a similar immunofluorescence-based approach, Karen and Hearing [72] showed that the number of protein VII foci in the nucleus steadily declined from 2 to 14 hpi. DNA replication was not required for loss of the protein VII foci, however blocking viral transcription either pharmacologically (alpha-amanitin) or through use of an E1A-deficient virus blocked the transition, once again suggesting that active viral transcription was required for release of VII from the Ad genome. 

Similarly conflicting results regarding the duration and level of association of protein VII with the viral genome have been shown using ChIP-based studies. While some studies have shown that protein VII is stably associated with the Ad DNA during early stages of infection [70,74], other studies showed a gradual decrease in VII association with the viral DNA over time [75,76,77]. During this same time period, there was a gradual increase in the association with histone H3 (and the other core histones) over most promoter and coding regions, although the binding levels of the histones was less than cellular chromatin [74]. Similar results were observed by Haruki *et al.* [75] who demonstrated the uneven distribution of VII along the Ad genome, with relatively low occupancy at the E1A promoter and relatively high occupancy at the major late promoter (MLP) at 6 hpi. The MLP only becomes active after viral DNA replication has initiated (~8–12 hpi), suggesting protein VII association may be more prolonged for regions of the Ad genome that are activated late in the virus lifecycle. Since it is very likely that the histones were binding directly to the DNA, these observations suggest that at least a portion of protein VII must be removed or remodeled to make space for binding of the histones to the viral DNA. Indeed, protein VII and H3 could be found bound to the same DNA molecules in re-ChIP experiments [74]. Protein VII may actually play a role in regulating early viral transcription, as pre‑wrapping plasmids in small quantities of purified protein VII led to higher levels of reporter gene expression compared to naked plasmid in transient transfection assays [74]. 

*In vitro* studies have shown that the protein VII-condensed DNA structure does not allow for efficient transcription [40,41], suggesting that the complex must be remodeled before efficient gene expression can occur. Again based on *in vitro* studies, three proteins have been identified that can remodel the VII-wrapped Ad genome: template activating factor Iβ (TAF-Iβ) (also known as SET [40]), TAF-II (NAP-1 [78]) and TAF-III (B23/nucleophosmin [79]). Of the three proteins, TAF-1β is the best characterized with respect to Ad DNA remodeling [70,75,80]. TAF-1β binds the VII‑wrapped DNA complex, which “opens” the viral DNA to allow access to nucleases and, presumably, transcriptional activators [41]. Whether remodeling by TAF-1β involves simple translocation or actual disassociation of VII from the DNA has yet to be determined. The observation that TAF-1β knockdown in cells has only a minor effect on Ad early gene expression suggests that accessory proteins may also be required for efficient transcription from the Ad template or that redundant mechanisms exist for remodeling [74].

Whether active transcription of the Ad DNA is required to mediate VII disassociation is also a subject of debate. Although some studies showed that VII appeared to remain associated with the viral DNA for longer periods of time when transcription elongation was inhibited [71,72], other studies did not see a difference [74,76]. It has been suggested that protein VII can recruit E1A to the viral DNA, and that E1A-mediated activation of transcription may lead to removal of protein VII [71,81]. Consistent with this, infection with an E1A-deficient virus prevented loss of the protein VII foci [71]. Again based on immunofluorescence analysis, release of protein VII could be achieved in the absence of E1A or other viral proteins, if the vector contained a strong heterologous promoter, such as the CMV promoter which is not reliant on E1A for expression [71]. Since only part of the AdWT genome is actively transcribed during early times of infection, it may be that protein VII is released from transcribed regions, but remains associated with the “late” regions until these regions are subsequently transcribed. This suggestion may also be consistent with the immunofluorescence-based studies, as this technique cannot easily distinguish between full- and partial-occupancy of VII on the viral DNA. 

## 7. Ad Vector DNA Associates with Cellular Histones in the Infected Cell Nucleus

Recent studies have established that Ad and its derivative vectors (E1-deleted, replication defective Ad or hdAd) associate with cellular histones early during infection [45,74,76,77]. Histones are detected on the Ad DNA within one hour of infection, and protein VII and histones can be found associated simultaneously with the same viral DNA fragment within the cell [74]. In general, deposition of cellular histones can occur through either a replication-coupled or replication‑independent mechanism, and the specific histone variants and chaperones involved vary for each mechanism [82]. Histone H3.1, which is expressed strictly during S-phase, is deposited on newly synthesized cellular DNA in a replication-coupled mechanism by the Chromatin Assembly Factor I (CAF-1) complex [83]. Conversely, the replacement histone variant H3.3, which differs from H3.1 by only 5 amino acids and is expressed throughout the cell cycle, is deposited through a replication-independent mechanism [82]. The histone chaperone involved in the deposition of H3.3 varies depending on the region of the chromosome. Within actively transcribed genes, the chaperone HIRA mediates H3.3 deposition, whereas on telomeres and pericentric DNA repeats, the H3.3 chaperone DAXX assists in the deposition [84,85,86]. Recent studies have suggested that the rapid deposition of H3.3 mediated by HIRA may be an evolved mechanism to protect “naked” DNA from damage [87]. 

In ChIP-based studies, hdAd, E1-deleted Ad [76] and wtAd [77] DNA associates with H3.3 as early as four hours post-infection, which suggests a replication-independent mechanism is responsible for the assembly of chromatin on Ad DNA. Recent work by Komatsu *et al.* [77] showing that knocking down of CAF-1 does not affect histone deposition on the Ad genome supports this idea [77]. Knockdown of HIRA reduced association of the hdAd DNA with H3, and also reduced Ad-mediated transgene expression [76], suggesting that assembly into chromatin is required for optimal gene expression. Given that H3.3 is also deposited on incoming Herpes Simplex Virus-1 DNA [88], the cell may utilize a common mechanism for processing incoming DNA originating from nuclear viruses.

AdWT DNA associates with all core histones (H2A-H2B and H3-H4) as early as one hour post‑infection [74,77]. Ad vector DNA is also found associated with all these nucleosomal proteins, and the DNA displays a classic nucleosomal laddering pattern upon micrococcal nuclease digestion [76], suggesting that the Ad DNA is wrapped into physiologically-spaced nucleosomes in the infected cell nucleus. Taken together, these studies suggest that assembly of Ad vector DNA into chromatin through deposition of histones and remodeling into nucleosomes are accomplished through a replication-independent mechanism, and this event is important in establishing optimal transgene expression.

## 8. Epigenetic Regulation of Ad Vectors

Assembly of hdAd DNA into chromatin is necessary for efficient expression of vector-encoded genes, and this event very likely contributes to their significant stability and efficacy *in vitro* and *in vivo* [27,65]. One of the advantages of hdAd is their large cloning capacity, which permits the use of large upstream regulator sequences, or even whole genomic loci, to permit tissue-specific gene expression [18,89,90]. Chromatin plays an important role in gene regulation [91,92], and proper placement of nucleosomes relative to the transcription start site of a gene is crucial for promoter fidelity [93,94]. The ability to incorporate large regulatory regions into hdAd coupled with its swift assembly into physiologically-spaced nucleosomes likely contributes to the maintenance of a faithful expression profile from these control elements when contained in hdAd.

As noted above, hdAd vector DNA must be designed to an optimal size in order to ensure physical stability of the capsid and genetic stability of the genome [30,42,43]. Thus, for small transgenes, additional non-coding “stuffer” DNA must be included in the vector in order to obtain an optimal size. However, the nature of this stuffer DNA can have a significant effect on function of the vector. A hdAd containing 22 kb of eukaryotic stuffer DNA expressed its transgene ~10-fold higher *in vitro* and *in vivo* compared to a vector with an identical expression cassette but containing stuffer DNA derived from prokaryotic DNA [44]. Neither of these vectors was subjected to CpG methylation and both vectors associated with histones to a similar degree. No evidence of heterochromatic methylation marks (*i.e.*, H3K9me2, H3K27me3) were observed, however hdAd-prok chromatin was markedly under-acetylated (a mark of transcriptionally inactive chromatin) [45]. This effect could be blocked through the use of a DNA insulator element or administration of trichostatin A (a histone deacetylase inhibitor), suggesting that a repressive chromatin structure assembled on the prokaryotic stuffer DNA and spread to the transgene. Cellular proteins Sp100 and DAXX were implicated in mediating this event [45], which is perhaps not surprising given that AdWT has evolved a specific mechanism to antagonize DAXX activity [95,96,97]. Indeed, a recent study has suggested that protein VI from the incoming virion may function to attenuate the action of DAXX, thus facilitating initiation of early gene expression [98]. 

A recent study has shown that virus-mediated activation of innate immune signaling can culminate in epigenetic down-regulation of expression from Ad vectors. Infection of MyD88^−/−^ mouse embryonic fibroblasts with a hdAd vector resulted in a higher level of transgene expression and was associated with an increased ratio of acetylated-H3K9 to methylated-H3K9 (*i.e.*, a tendency towards “active” chromatin configuration), compared to hdAd-infected wildtype mouse embryonic fibroblasts [99]. This observation suggests that engagement of toll-like receptors (such as TLR9 [100]) by Ad vector infection, and subsequent activation of MyD88-mediated signaling cascades, can lead to epigenetic silencing of the vector DNA. Thus, this study uncovered an innate pathway designed to epigenetically regulate expression of genes located on invading DNA.

Ad-encoded proteins can also actively modulate the activity of certain cellular chromatin modifying complexes. For example, the early region 4 open reading frame 4 (E4orf4) protein was shown to bind AcfI and modulate the activity of the ACF chromatin remodeling complex [101]. E4orf4 appears to recruit protein phosphatase 2A (PP2A) to the ACF complex (and thus to chromatin), where PP2A may dephosphorylate uncharacterized substrates and alter local chromatin structure. It is thought that this interaction may contribute to E4orf4-induced caspase-independent, non-classical apoptosis at late times of AdWT infection [101,102,103]. Since first-generation Ad vectors retain the E4 region, it is therefore possible that expression of E4orf4 may in part contribute to acute or chronic toxicity noted for E1-deleted vectors [5]. 

## 9. Conclusions and Future Perspectives

Ad vectors are one of the most commonly used systems to deliver genes to mammalian cells. In the nucleus, the viral DNA must conform to the rules of the host environment, and many studies have shown that histones and proper chromatin configuration are as important to Ad as it is to the host DNA. Histones deposited on the Ad DNA can adopt an “active” euchromatic state or an “inactive” heterochromatic state. In the case of more advanced generations of Ad vectors, such as the hdAd, designing the vector such that it resembles a segment of the chromosome (e.g., using genomic loci‑based expression constructs or large, non-coding stuffer elements derived from the human genome) allows these vectors to retain high-level expression for many years in mice, rats, dogs and non-human primates. Improving our understanding of the mechanism of assembly of Ad DNA into chromatin, and the cellular proteins involved in modulating this nucleoprotein structure, will undoubtedly improve our ability to design the next generation of Ad vector exhibiting true tissue-specific transgene regulation, optimal safety, and expression. Similarly, uncovering aspects of Ad vector design that permit the vector to remain undetected in the host cell nucleus should improve the function and persistence of the vector DNA within the transduced cell. For AdWT, there are a few obvious areas that require further study, such as whether protein VII is truly maintained and/or required on certain regions of the Ad genome during the early phase of viral replication, and how its association is affected by active transcription. Since the wildtype virus must transition from association with protein VII to histones and back to protein VII for packaging of the DNA into progeny virions, the cellular protein(s) that facilitate these events have yet to be determined. Finally, studying how viruses and vectors have adapted to function with the context of a mammalian nucleus will undoubtedly provide novel insight into how chromatin structure impacts host cell DNA function. 
